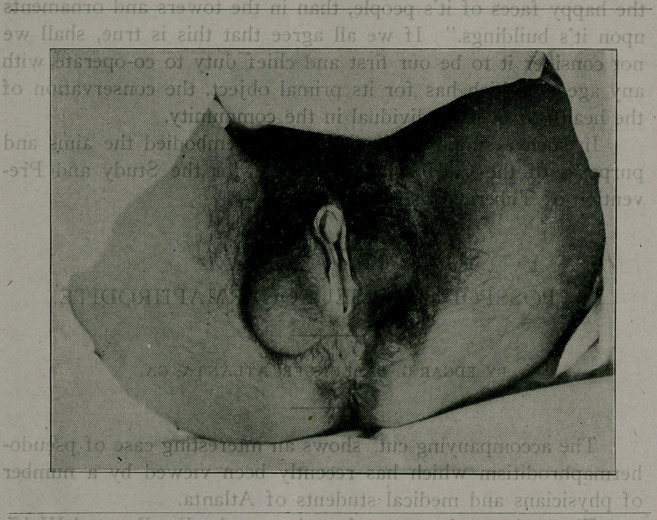# Hyposspodias—Pseudohermaphrodite

**Published:** 1908-04

**Authors:** Edgar G. Ballenger

**Affiliations:** Atlanta, Ga.


### HYPOSSPODIAS—PSEUDOHERMAPHRODITE.

BY EDGAR G. BALLENGER, ATLANTA, GA.

The accompanying cut shows an interesting case of pseudohermaphroditism which has recently been viewed by a number of physicians and medical students of Atlanta.

The patient, who was referred to me by Dr. Bernard Wolff, for demonstration at the Fulton County Medical Society, gives the following history: Miss (?) S. H., aged 39 born in North Georgia, at birth was more like a girl than boy and consequently was dressed like a girl, and has continued this form of dress although there are no female sexual organs, the uterns and ovaries not being palpable.

About the age of 17 or 18 the patient claims to have noted a slight enlargement of the breast, and that menstruation was observed three or four times soon after; this was not accompanied by any pain. There is more sexual desire for men than for women. Sometimes has "spending dreams," dreaming of coitus with a man. Less frequently dreams of having intercourse with woman, but something always seems to intefere with or interrupt the consummation of the act before organism is reached. Masturbation is indulged in several times a week—this may be done either as a male or female, there being little difference in the sensation. The penis is about one and one-half inches in length, but when erect becomes two or three times this size. The sinus forming the pseudo-vagina is about three inches in depth. The urethral canal opens just within it on the anterior wall. The testicles are well developed and situated on each side of the labia in a rather loose pouch of skin or scrotum. The epididymes are of normal size and the right one contains a small nodule which is the remains of a previous epididymitis. (There is no history of specific infection.) By rectal examination the prostate could be felt as two nodules—one on each side of the urethra. The one on the right is round and about the size of a hazelnut; that on the left is flattened and apparently a little smaller. There seems to be no connection between these two lobes. No uterus tubes or ovaries could be detected by careful rectal examination.

The patient is tall and slender; no beard, but long hair of medium texture; the voice reminds one of a woman rather than a man. The general health is good, and there is nothing unusual or of interest in the past history.

## Figures and Tables

**Figure f1:**